# Influence of decontamination strategies on the bond strength of a universal adhesive bonded to hemostatic agent–contaminated dentin

**DOI:** 10.1016/j.jobcr.2026.101557

**Published:** 2026-11-05

**Authors:** Ria Sharma, Rajni Nagpal, Udai Pratap Singh, Garima Tyagi, Megha Nagpal, Sakshi Saumya

**Affiliations:** a Department of Conservative Dentistry and Endodontics, Kothiwal Dental College and Research Centre, Moradabad, Uttar Pradesh, India; b Department of Conservative Dentistry and Endodontics, Kothiwal Dental College and Research Centre, Moradabad, Uttar Pradesh, 244001, India

**Keywords:** Aluminum chloride, Decontamination, Dental bonding, Hemostatics, Shear strength, Universal adhesives

## Abstract

**Objective(s):**

To evaluate the effect of aluminum chloride (AlCl_3_)–based hemostatic agent contamination and various decontamination protocols on the shear bond strength (SBS) of a universal adhesive applied in self-etch mode, and to assess resin–dentin interface adaptation using scanning electron microscopy (SEM).

**Materials and methods:**

Eighty-four extracted human molars were prepared and randomly assigned to seven groups (n = 12): control, contaminated, and five decontamination groups (water, sodium hypochlorite [NaOCl], chlorhexidine [CHX], phosphoric acid, and ethylenediaminetetraacetic acid [EDTA]). Following contamination and respective surface treatments, Prime & Bond Universal adhesive was applied in self-etch mode. Composite cylinders were bonded to dentin surfaces, and SBS was measured. One specimen from each group was subjected to SEM analysis to evaluate the resin–dentin interface. Data was analyzed using one-way analysis of variance (ANOVA) and Tukey's post hoc test (*p* < 0.05).

**Results:**

Contamination with the hemostatic agent significantly reduced SBS (*p* < 0.001). Decontamination with NaOCl, phosphoric acid, and CHX restored bond strength to values comparable to the control group (*p* > 0.05). In contrast, water and EDTA groups showed significantly lower SBS than the control (*p* < 0.05). SEM analysis supported these findings, demonstrating improved interfacial adaptation in NaOCl, phosphoric acid, and CHX-treated groups.

**Conclusion(s):**

AlCl_3_-based hemostatic agent contamination adversely affected bonding performance. Decontamination with NaOCl, phosphoric acid, and CHX effectively restored bond strength and interfacial integrity, whereas water and EDTA were less effective.

## 1 Introduction

Ensuring a precise control over blood and gingival crevicular fluid is very crucial in restorative procedures, particularly when dealing with composite restorations for class V cavities that involve subgingival margins. A dry operating field is imperative for successful composite restorations, as contamination with blood and moisture can adversely affect the formation of a flawless hybrid layer and jeopardize the bond strength between resin-based restorative materials and tooth structure.^1^ These contaminants have the potential to infiltrate the dentinal tubules, thus disrupting adhesive diffusion into the dentinal tubules and potentially compromising the overall quality of adhesion in dental restorative procedures.^2^

Although rubber dam is the optimal method of isolation, the application of a topical hemostatic agent remains the prevalent choice among clinicians to manage bleeding and reduce gingival fluid flow during dental procedures.^3^ The use of a retraction cord impregnated with an appropriate hemostatic agent effectively controls gingival bleeding and sulcular fluid.^4^ The most commonly used hemostatic agents are AlCl_3_ and ferric sulfate [Fe_2_(SO_4_)_3_]. Aluminum compounds, commonly used at concentrations of 20%–25%, promote hemostasis by precipitating proteins, constricting blood vessels, and removing tissue fluid to provide a dry operative field.^5^

However, hemostatic agents may lead to a significant reduction in the bond strength of adhesive to dentin.^6^ Prior research has suggested that following water rinsing, the hemostatic agent still persists on the tooth surface, resulting in a notable decline in the bond strength, particularly with self-etch adhesive systems.^7^, ^8^, ^9^, ^10^ Even after water rinsing, the residual aluminium and chloride ions have been identified on the dentin surface, and the bond strength of universal adhesive in self-etch mode is thus found to be compromised due to hemostatic agent contamination.^11^ Furthermore, these residual particles of hemostatic agents may impede the flow of resin monomers into the dentinal tubules, thereby detrimentally impacting bond strength.^6^

In response to these apprehensions, effective protocols for decontaminating inadvertently hemostatic agent contaminated dentin are of paramount importance. Prior literature puts forward quite a few approaches in order to enhance the effective dissolution of various minute remnants of the hemostatic agent from the dentin surface including rinsing with water and air drying or rinsing with agents such as NaOCl, CHX or with EDTA post contamination. The application of these agents has been done as dentin pretreatment protocol prior to the application of bonding agent and subsequent resin restoration. 12, 13, 14, 15, 16, 17.

Although many investigators have tested the effect of hemostatic agent contamination and various decontamination protocols on the bonding of etch and rinse adhesive, very few studies have comparatively evaluated the effect of multiple clinically feasible decontamination protocols together, specifically with universal adhesives applied in self-etch mode.^18^, ^19^, ^20^, ^21^ Hence, the objective of this in vitro investigation was to comparatively assess the effect of different decontamination procedures on the SBS between a universal adhesive and dentin following contamination with an AlCl_3_–based hemostatic agent.

## 2 Materials and methods

The current in-Vitro experimental study was reviewed and approved by the Ethical Committee of Kothiwal Dental College and Research Centre, Moradabad, Uttar Pradesh, India (reference no: KDCRC/IERB/02/2024/03). Following approval from the Institutional Ethics Committee, 84 recently extracted human molars were obtained for the study. Sample size estimation was performed using G*Power software (version 3.1.9.7), which yielded an effect size of 1.564 with an actual statistical power of 95.55%. Based on these parameters, the required sample size was calculated to be 12 specimens per group. Accordingly, a total of 84 specimens were included and randomly allocated into seven study groups. The teeth had been extracted for orthodontic or periodontal reasons no more than three months before the commencement of the experiment. Teeth with developmental anomalies, restorations, or cracks were excluded from the study.

Human molars were selected because they provide a broad, relatively flat coronal dentin surface, allowing standardized specimen preparation and an adequate bonding area for shear bond strength testing. Their larger dentin surface also minimizes variability during specimen preparation compared with premolars or anterior teeth.

### 2.1 Sample preparation

84 human adult molars were collected and cleaned of debris and calculus and stored in 0.1% thymol solution and kept in a refrigerator at a temperature of about 4°C. The collected teeth were completely cleaned using ultrasonic scaler followed by thorough cleaning with pumice slurry and rubber prophylactic cups rotating at slow speed in the contra-angled micromotor handpiece. Molars were prepared to reveal flat coronal dentin. All the teeth were flattened occlusally with a slow-speed diamond saw (Isomet, Buehler Ltd., Lake Bluff, IL, USA) under vigorous water irrigation to expose the flat coronal dentin. A standardized smear layer was created with 600 grit silicon - carbide (SiC) paper. The samples were embedded in an auto polymerizing resin (Ortho-Jet; Lang Dental Manufacturing Co. Inc., Wheeling, IL, USA) at the level of cemento-enamel junction with long axis perpendicular to the acrylic resin surface. They were then randomly divided into seven groups based on surface pre-treatments (n = 12):

**Group I: Control group**: no contamination: Dentin surface was air dried to remove excess moisture.

**Group II: Hemostatic agent + air drying**: Hemostatic agent containing AlCl_3_ (Viscostat Clear, Ultradent) was applied on dentin surface for 2 min and then air dried.

**Group III: Hemostatic agent + water rinsing**: Hemostatic agent was applied on dentin surface for 2 min, rinsed with high pressure water spray for 60 s and then air dried for 5 s to remove excess moisture.

**Group IV: Hemostatic agent + chlorhexidine**: Hemostatic agent was applied on dentin surface as in group III. The tooth surfaces were then rinsed with 2% CHX solution for 1 min, rinsed with water for 60 s, and air dried for 5 s to remove excess moisture.

**Group V: Hemostatic agent + sodium hypochlorite**: Hemostatic agent was applied on dentin surface as in group III. The tooth surfaces were then rinsed with NaOCl for 1 min, rinsed with water for 60 s and air dried for 5 s to remove excess moisture.

**Group VI: Hemostatic agent + EDTA:** Hemostatic agent was applied on dentin surface as in group III. 17% EDTA solution was then administered for 1 min, rinsed for 60 s and then air dried for 5 s to remove excess moisture.

**Group VII: Hemostatic agent + phosphoric acid**: Hemostatic agent was applied on dentin surface as in group III. 36% phosphoric acid was then administered for 15 s, rinsed for 30 s and air dried to remove excess moisture.

### 2.2 Restorative procedures

These surface treatments were followed by Universal adhesive (Prime & Bond universal, Dentsply Sirona) application according to manufacturer's instructions in all the groups. Transparent plastic tubes (Medrop Nasogastric tube) with 3 mm internal diameter and 2 mm in height and thickness of 0.5 mm were pre sliced and were placed perpendicular to previously bonded dentin surfaces for composite resin buildup. A microhybrid resin composite (Spectrum® Universal Microhybrid composite resin, Dentsply Sirona, Konstanz, Germany) was filled into the pre-sliced tubes. After composite placement each specimen was light-cured for 20 s at 600 mW/cm^2^ with a Bluephase® LED light-curing unit (Ivoclar Vivadent, Schaan, Liechtenstein). The plastic tubes were delicately sliced and carefully removed with a number 11 surgical blade after light curing. Specimens were then stored in distilled water at 37°C for 24 h for completion of polymerisation before testing. Specimens were thermocycled in a water bath for 30,000 cycles between 5°C and 55°C with a 30-s dwell time and a 30-s transfer time to simulate the conditions of the oral cavity.

### 2.3 Determination of SBS

12 samples from each group were subjected to shear bond strength assessment under Universal testing machine (UTM) after 24 h storage in distilled water to allow water sorption and complete polymerisation. The specimens were tested in shear mode under UTM at crosshead speed of 1 mm/min. Load was applied until restoration failure. Bond strength was calculated in Mega Pascal (MPa) using maximum force in Newton (N) applied per unit area (mm^2^).

### 2.4 Fractographical analysis

All the debonded specimens were analyzed under SEM (ALCO® Stereo Zoom Microscope, ALCO Instruments, India) at 10 X magnification to classify the type of bond failure, either as adhesive (a), cohesive (c) & mixed (m).

### 2.5 SEM evaluation

One sample from each group was subjected to SEM evaluation. The restored samples were sectioned longitudinally employing slow-speed diamond saw under continuous water cooling to obtain flat, uniform sections and polished using progressively finer silicon carbide abrasive papers up to #1200 grit under running water. Following polishing, specimens had been decalcified in 6N HCl for 30 s and rinsed with distilled water. Ten minutes of deproteinization with 1% NaOCl and a final rinse in distilled water was followed. Specimens had then been dehydrated in ethanol at 25, 50, 75, & 95% for 20min, then 100% for 1 h. The dehydrated specimens were then subjected to critical point drying for 30 min. After drying, a platinum-palladium alloy was sputter-coated onto the samples using a sputter coater (JFC-1600 Auto Fine Coater; JEOL Ltd., Tokyo, Japan). SEM (JSM-6490, JEOL Ltd., Tokyo, Japan) had been employed for investigating specimens to evaluate the resin–dentin interface morphology.

### 2.6 Statistical methods

The data was entered into Microsoft Excel XP software program. Statistical analysis was done by Statistical Package for the Social Sciences (SPSS) software package (SPSS 16 Inc, Chicago IL, USA). The normality of data was tested by Shapiro Wilk's test. The values obtained was statistically analyzed and to compare the parameters between groups and with in groups for normal data parametric test one way ANOVA followed by Tukey's HSD test, was used among the study population. The level of significance and confidence interval was 5% and 95% respectively, i.e. *p* < 0.05.

## 3 Results

In the present study, all experimental groups exhibited variations in mean SBS when subjected to different surface pretreatments prior to bonding. The distribution of SBS values in all groups was first assessed for normality using the Kolmogorov–Smirnov and Shapiro–Wilk tests. All groups exhibited *p* values > 0.05 in both tests confirming that the data followed a normal distribution and validating the use of parametric statistical tests. One-way ANOVA revealed a statistically significant difference among the seven groups (*p* < 0.001), indicating that the type of surface pretreatment had a significant effect on the mean SBS values.

The control group (Group I), exhibited a mean bond strength of 38.56 MPa, representing the expected baseline performance of the adhesive system (Table 1, Table 2). In contrast, the hemostatic agent–contaminated group (Group II) showed a significant reduction (20.35 MPa), demonstrating that contamination with AlCl_3_-based hemostatic agents significantly reduced bond strength. (p < 0.001)

**Table 1:** Descriptive statistics of shear bond strength (MPa) of different groups.

Group	N	Mean	Std. Deviation	Std. Error	95% Confidence Interval for Mean		Minimum	Maximum
					Lower Bound	Upper Bound		
Group I	12	38.5600	3.37246	0.97355	36.4172	40.7028	32.95	43.26
Group II	12	20.3500	2.11426	0.61033	19.0067	21.6933	16.64	24.32
Group III	12	22.8900	2.52978	0.73028	21.2827	24.4973	17.55	26.22
Group IV	12	35.2300	3.17945	0.91783	33.2099	37.2501	30.80	41.11
Group V	12	45.2967	3.92291	1.13245	42.8042	47.7892	40.16	53.49
Group VI	12	19.6400	3.07549	0.88782	17.6859	21.5941	15.55	26.83
Group VII	12	38.6400	2.26144	0.65282	37.2031	40.0769	34.52	42.82

The values are mean ± Std. Deviation. Std. Deviation, standard deviation; Std. Error, standard error.

**Table 2:** Multiple comparisons of means of shear bond strength (MPa) between two different groups by Tukey HSD test**.**

Groups	Mean	*p,* significance	*p,* significance	One-way ANOVA
Group I	—	Versus Group II: <0.001† Versus Group III: <0.001† Versus Group IV: 0.104	Versus Group V: <0.001† Versus Group VI: <0.001† Versus Group VII: 1.000	*p* < 0.001
Group II	—	Versus Group III: 0.372 Versus Group IV: <0.001† Versus Group V: <0.001†	Versus Group VI: 0.997 Versus Group VII: <0.001†	
Group III	—	Versus Group IV: <0.001† Versus Group V: <0.001†	Versus Group VI: 0.121 Versus Group VII: <0.001†	
Group IV	—	Versus Group V: <0.001† Versus Group VI: <0.001†	Versus Group VII: 0.089	
Group V	—	Versus Group VI: <0.001†	Versus Group VII: <0.001†	
Group VI	—	Versus Group VII: <0.001†	—	
Group VII	—	—	—	

The values represent pairwise multiple comparisons of shear bond strength using the Tukey HSD test following one-way ANOVA. ANOVA, analysis of variance; HSD, honestly significant difference. †Statistically significant difference (*p* < 0.05).

Among the decontaminated groups, distinct trends were observed, water-rinsed group (Group III) recorded a mean SBS of 22.89 MPa, significantly lower than the control (p < 0.001) and not significantly different from the hemostatic agent group. CHX-treated group (Group IV) demonstrated improved SBS (35.23 MPa), not significantly different from the control (p = 0.104) but significantly higher than the hemostatic agent and water-rinsed groups. NaOCl group (Group V) exhibited the highest mean bond strength (45.30 MPa), significantly higher than the control (p < 0.001) and all other experimental groups indicating superior cleaning efficacy and enhanced adhesive interaction.

EDTA-treated group (Group VI) showed the lowest mean SBS (19.64 MPa) among all pretreatments, significantly lower than the control (p < 0.001) and NaOCl (p < 0.001), but statistically similar to the hemostatic agent group (*p* = 0.997). Phosphoric acid group (Group VII) demonstrated a mean SBS of 38.64 MPa, comparable to the control (p = 1.000) and significantly higher than both the hemostatic agent and EDTA (*p* < 0.001) groups.

### 3.1 Fractographical analysis

Mixed failure was found to be the most predominant in most groups (Control, HA + CHX, HA + NaOCl, HA + Phosphoric), accounting for about 50–58%. Adhesive failure was highest in HA + Air and HA + EDTA (∼66–67%), indicating weaker bonding in these groups, with HA + Water also showing relatively high adhesive failure (∼58%). Cohesive failure was minimal or absent in most groups, remaining the least common overall. Overall, the results indicate a general predominance of mixed failures, except in air- and EDTA-treated groups where adhesive failure dominated (Fig. 1).

**Fig. 1:**
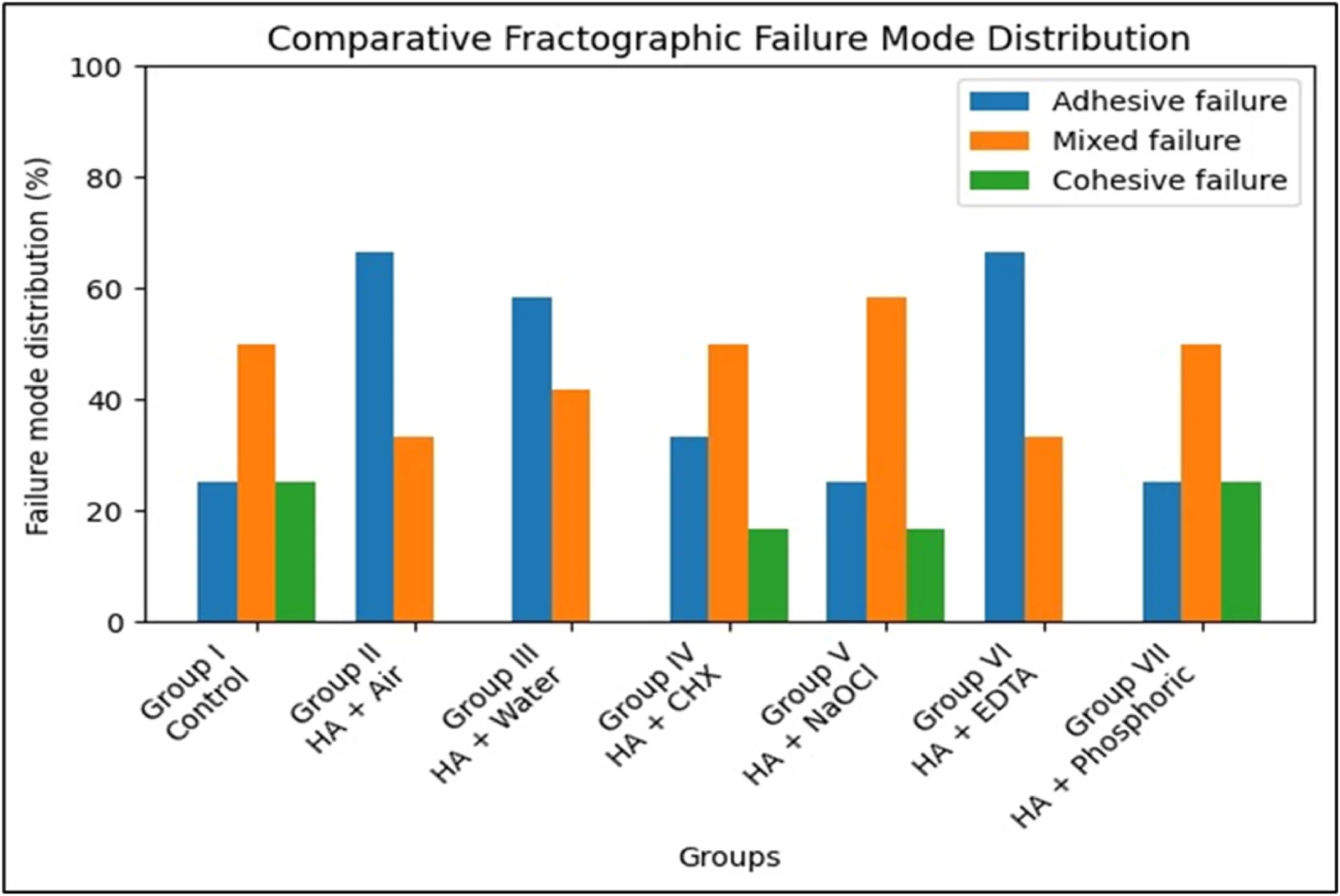
Failure modes after the shear bond strength test.

### 3.2 SEM evaluation

Fig. 2(a–g) display SEM images of resin–dentin interface for all groups. Good interfacial adaptation was seen in control group as well as when decontamination was performed with NaOCl, CHX and phosphoric acid whereas water rinsing and EDTA did not provide satisfactory interfacial seal.

**Fig. 2:**
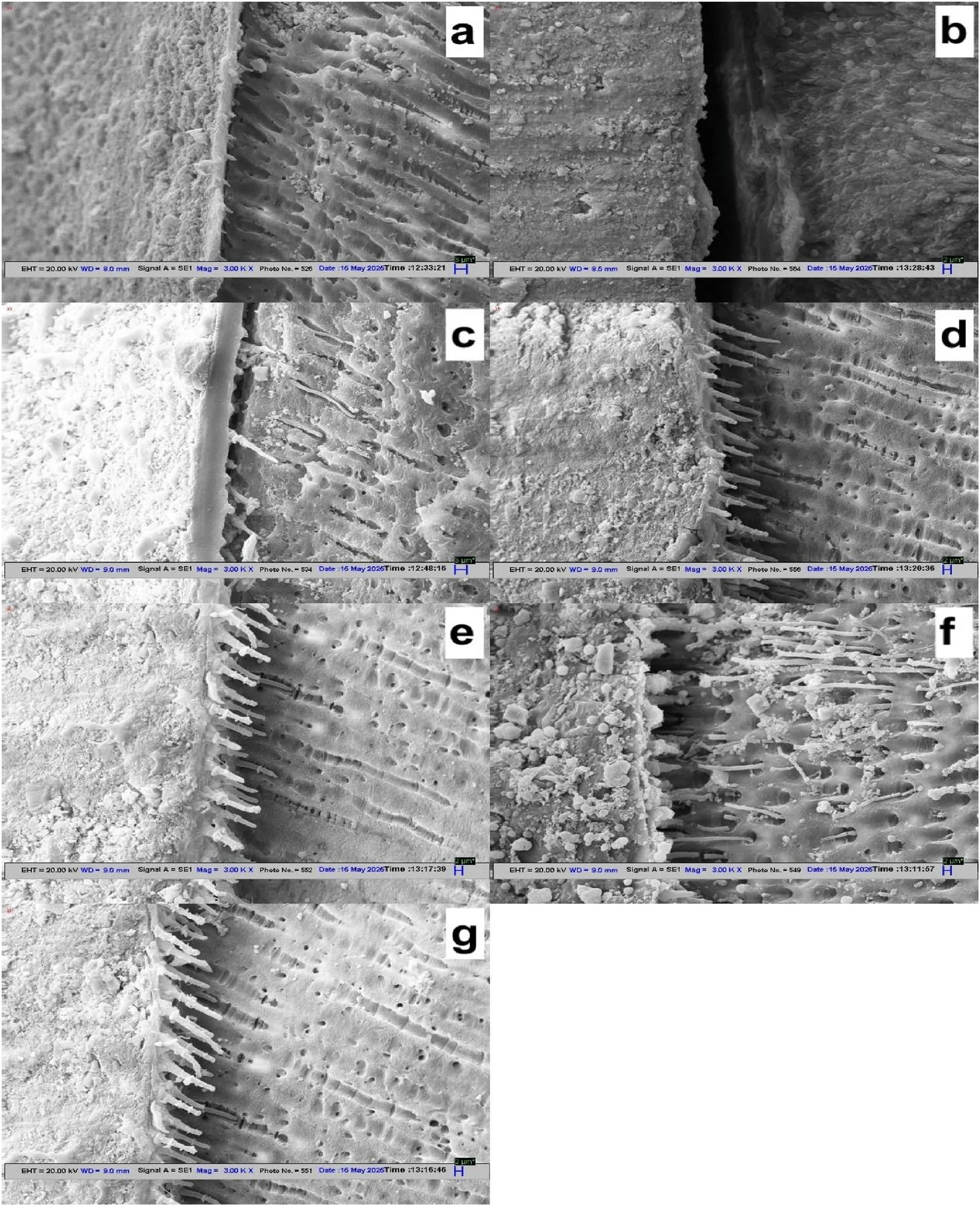
SEM photomicrographs at 3000× of adhesive-dentin interface of: a) Group I (control); b) hemostatic agent contamination (group II); decontamination protocols: c) water rinsing (group III); d) chlorhexidine, CHX (group IV); e) sodium hypochlorite, NaOCl (group V); f) ethylenediaminetetraacetic acid, EDTA (group VI); g) phosphoric acid (group VII).

## 4 Discussion

The bonding of composite to tooth structure relies on a combination of micromechanical interlocking and chemical interaction between functionalized resin monomers and hydroxyapatite, making it a technique-sensitive procedure.^22^ Due to this sensitivity, various factors can influence or interfere with the bonding process, potentially compromising the quality of the final restoration.^23^

Successful adhesion requires careful tooth surface preparation, demineralization using conditioning agents, and strict control of the operative environment. However, complete isolation is not always achievable in routine dental practice.^23^ Situations such as operative sites near or at the gingival margin, deep proximal cavities, uncooperative patients, recent surgical areas, or teeth with cervical defects or malalignment can make isolation difficult.^24^ The presence of saliva, blood, water, gingival crevicular fluid or using a retraction cord with hemostatic agent for effectively controlling gingival bleeding and sulcular fluids can significantly impair the bonding efficiency of adhesive systems to dentin.^25^ Therefore, decontamination procedures aimed at removing any residual contaminants should be taken into account.

To achieve adequate moisture control during restorative procedures at the gingival margin, AlCl_3_ and ferric salt hemostatic agents are frequently used to control gingival bleeding and sulcular fluid.^5^^,^^26^ However, their acidic nature and protein-precipitating action may leave residues on the prepared dentin surface that alter smear-layer morphology, occlude tubules, and hinder resin monomer infiltration; collectively these effects can reduce bond strength of resin-based restoratives.^5^^,^^22^

AlCl_3_ has minimal systemic effects and produces vasoconstriction. Ferric sulfate also functions as a vasoconstrictor and causes coagulation of blood proteins when they interact with sulfate and iron ions under acidic conditions. These coagulated proteins create a network that effectively blocks the capillary openings^27^ In laboratory and clinical reports AlCl_3_ tends to cause less reduction in adhesive bond strength than Fe_2_(SO_4_)_3_.^7^^,^^10^^,^^14^ Ferric salts also produce darker staining/discoloration of dentin because iron ions penetrate and precipitate in dentinal tubules, whereas aluminum salts are less prone to cause this permanent staining.^27^ Clinically, Fe_2_(SO_4_)_3_ has been associated with more postoperative sensitivity and greater tissue irritation in some reports, while AlCl_3_ is generally considered effective for bleeding control with fewer aesthetic and tissue-reaction drawbacks.^28^ Therefore, hemostatic agent containing AlCl_3_ was used in this study.

In the current study, the universal adhesive was used in the self-etch mode. Universal adhesives contain the functional monomer 10-MDP, which accounts for their effective etching capability and strong bonding to various substrates.^29^ When applied as a single-step self-etch adhesive, they help maintain the moisture of the demineralized dentin and prevent collagen fibril collapse. Moreover, using these systems in the self-etch mode reduces technique sensitivity and makes them particularly suitable for areas where moisture control is challenging, such as posterior regions.^30^ For this reason, the self-etch approach was selected. A growing body of in-vitro evidence shows that hemostatic contamination most often has a larger adverse impact on self-etch adhesive applications than on etch-and-rinse (ER) approaches.^21^^,^^31^^,^^32^ In particular, studies using universal adhesives applied in self-etch mode report marked reductions in microtensile or SBS after contamination, whereas ER application (re-etching prior to adhesive) tends to mitigate or eliminate that effect.^16^^,^^21^^,^^32^ This etching-mode dependence was directly demonstrated by Pratabsingha et al. in their study, where universal adhesive micro-tensile bond strength (μTBS) to hemostatic agent contaminated dentin was significantly impaired in self-etch mode but could be restored by etch-and-rinse mode or EDTA pretreatment.^32^ Ajami et al., Bahari et al. and Kuphasuk et al. also reported reduced bond strength of self-etch adhesives after hemostatic agent contamination of dentin.^7^^,^^14^^,^^33^

Systematic evidence supports the notion that any contamination (blood, saliva, or hemostatic agents) commonly reduces dentin bond strength and that the effectiveness of decontamination varies across methods. The recent systematic review by Bourgi et al. synthesized 62 in-vitro studies and concluded that re-etching, targeted chemical cleansers, and reapplication strategies are among the interventions most consistently reported to reverse contamination-induced bond loss, whereas simple rinsing alone is frequently insufficient.^22^

Our experimental results mirror these published patterns. The contaminated group (Group II) showed a large reduction in mean SBS (≈20.35 MPa) compared with the uncontaminated control (≈38.56 MPa), consistent with prior demonstrations that AlCl_3_ -based agents reduce resin infiltration through protein coagulation and surface precipitation.

Among the decontamination strategies tested in our study, NaOCl produced the highest mean SBS, exceeding even the control. Mechanically, NaOCl has a solvent action on organic debris, and it also acts as a deproteinizing agent modifying the surface of dentinal substrate. NaOCl's proteolytic action dissolves organic residues and denatured collagen, increases surface wettability, and can improve resin penetration and tag formation — effects also observed in other in-vitro investigations where NaOCl improved SBS after ViscoStat Clear (AlCl_3_) contamination.^34^^,^^35^ Faizal et al. reported that, in a self-etch adhesive system, dentin contaminated with a hemostatic agent and subsequently decontaminated with NaOCl exhibited the highest SBS, followed by the air-dried and water-treated groups.^20^

CHX restored bond strength nearly equivalent to control group in our findings. This is biologically plausible for two reasons: (1) CHX can help remove or disperse some contaminant residues when used as a rinse/cleaner in decontamination protocols, and (2) CHX is a potent inhibitor of dentin matrix MMPs and cathepsins, thereby helping preserve the collagen scaffold of the hybrid layer and improving long-term bond durability. Multiple in-vitro studies and reviews have demonstrated CHX's protective effect on bond degradation.^36^ The cationic properties of CHX may contribute to its ability to remove certain components of the hemostatic agent. It was revealed that CHX was unable to eliminate aluminum ions from the affected surface, whereas chloride ions were completely removed following CHX rinsing. This is likely because the strong positive charge of CHX allows it to interact with chloride ions and form a soluble complex that can be rinsed away. However, CHX does not appear sufficiently cationic to displace aluminum from the stable compound Al(OH)_2_H_2_PO_4_.^32^ Sharafeddin et al. reported that the use of CHX after the removal of hemostatic agent decreased the bond strength to dentin, this could be attributed to different bonding agents used.^10^ Therefore, is has been seen that there have been different opinions regarding the effectiveness of CHX as a decontaminant for hemostatic agent contaminated dentin. In our study, the bond strength of universal adhesive used in self-etch mode to hemostatic agent contaminated dentin was re-instated by CHX to levels almost equal to control but not as strongly as NaOCl.

Phosphoric acid re-etching (Group VII) returned SBS to a level essentially matching the control (≈38.64 MPa). Earlier studies have shown that when dentin is contaminated with AlCl_3_, the material can interfere with hydroxyapatite demineralization. This occurs through a substitution reaction in which calcium in hydroxyapatite is replaced by aluminum, leading to the formation of insoluble Al(OH)HPO_4_, a compound that is more resistant to acidic dissolution. Because self-etch primers are much less acidic than phosphoric acid, they are unlikely to effectively eliminate aluminum residues or penetrate deeply into the dentinal tubules. In contrast, the stronger acidity of phosphoric acid used in the etch-and-rinse technique allows sufficient demineralization of dentin and facilitates the removal of remaining surface contaminants. Therefore, phosphoric acid in the ER mode can act as an effective cleaning agent for dentin contaminated with AlCl_3_.^16^ Re-etching removes superficial residues and re-exposes mineralized dentin, increasing surface energy and micromechanical interlocking. Several experimental papers report phosphoric acid as an effective and practical method to recover bonding after hemostatic contamination.^37^

By contrast, water rinsing alone (Group III) did not restore SBS (≈22.89 MPa), consistent with multiple reports indicating that water rinsing often cannot displace tightly bound precipitates of aluminum or ferric salts and is therefore insufficient in many contamination scenarios.^7^^,^^38^ Multiple investigations have shown that simple water rinsing is insufficient to completely remove hemostatic agents from the tooth surface. Residual material can persist and lead to a marked reduction in bond strength, a problem particularly evident with self-etch adhesive systems.^10^^,^^16^^,^^18^

EDTA (Group VI) produced the lowest SBS in our data (≈19.64 MPa). Although EDTA is an effective chelating agent that can remove smear layer and certain ionic residues, its action may leave a demineralized collagen matrix that is not optimally infiltrated by the adhesive.^39^^,^^40^ Notably, Pratabsingha et al. found that EDTA rinsing (17%) restored μTBS in their protocol, indicating that EDTA can be effective when used with specific timing/concentrations — but EDTA results are protocol-sensitive and may be detrimental if misapplied.^32^ The study by Mempel et al. investigated whether EDTA could effectively decontaminate dentin surfaces contaminated with hemostatic agents—ViscoStat (ferric sulfate) and ViscoStat Clear (AlCl_3_) before bonding with universal adhesives in self-etch mode.^13^ Results showed that EDTA treatment failed to improve or restore the bond strength for either adhesive and in some cases further weakened adhesion. This reconciles our findings with the literature: EDTA can be a restorative strategy in some hands/protocols but may underperform or damage bonding in others.

Despite the favorable outcomes of this study, several limitations should be acknowledged. First, SBS testing was employed rather than μTBS evaluation. Although SBS is a simple, reproducible and widely accepted method for assessing adhesive performance, it generates a non-uniform stress distribution at the adhesive interface and may therefore be less sensitive than μTBS in detecting subtle differences in bond strength. In addition, failure during SBS testing may occur outside the adhesive interface, potentially influencing the measured bond strength values. Second, this investigation was conducted under in vitro conditions, which cannot fully reproduce the complex oral environment. Factors such as saliva contamination, pulpal pressure, occlusal loading, thermal fluctuations, enzymatic degradation, pH changes, and intraoral ageing may influence the long-term durability of the adhesive interface and were not completely simulated despite thermocycling. Furthermore, only sound coronal dentin was evaluated, therefore, the findings may not be directly applicable to caries-affected, sclerotic, or deep dentin, which differ considerably in their structural and mechanical characteristics. Another limitation is that only one commercially available aluminum chloride–based hemostatic agent and one universal adhesive applied in the self-etch mode were investigated. Consequently, the results cannot be directly extrapolated to other hemostatic formulations, adhesive systems, or bonding strategies, such as the etch-and-rinse approach. Finally, the SEM analysis was qualitative and performed on one representative specimen from each group; therefore, quantitative assessment of the hybrid layer and resin tag formation was beyond the scope of the present investigation. Future studies should evaluate different adhesive systems, hemostatic agents, decontamination protocols and ageing conditions to validate and extend the present findings. Clinically, when contamination occurs at subgingival margins or in situations where rubber-dam isolation is not feasible, active chemical decontamination using sodium hypochlorite or phosphoric acid re-etching should be preferred over water rinsing alone, while chlorhexidine may serve as an adjunct to enhance long-term preservation of the resin–dentin interface.

## 5 Conclusions

Among the tested decontamination methods, NaOCl achieved the highest recovery of bond strength of universal adhesive applied in self-etch mode to hemostatic agent contaminated dentin, restoring and even surpassing control. CHX and Phosphoric acid re-etching improved the bond strength and returned it to near-control levels, while water rinsing had no beneficial effect. EDTA application further compromised the bonding performance. Overall, the findings underscore that simple rinsing is inadequate and that active chemical cleansing or re-etching is necessary to reverse the detrimental effects of AlCl_3_ contamination. Further research is still needed to evaluate these protocols under clinically simulated conditions and with varying adhesive systems.

## Human and animal rights statement

This study did not involve any human participants or animals. The research was conducted as an in vitro experimental study using extracted human teeth.

## Institutional review board statement

Institutional Ethical Committee Review Board (Approval No. KDCRC/IERB/02/2024/03), Kothiwal Dental College and Research Centre, Moradabad, Uttar Pradesh, India.

## Funding

Self-Funding

## Sources of funding

Nil.

## Declaration of competing interests

The authors declare that they have no known competing financial interests or personal relationships that could have appeared to influence the work reported in this paper.
